# The sleep-feeding conflict: Understanding behavioral integration through genetic analysis in Drosophila

**DOI:** 10.18632/aging.100181

**Published:** 2010-07-27

**Authors:** Daniel M. McDonald, Alex C. Keene

**Affiliations:** Department of Biology, New York University, New York, NY 10003, USA

**Keywords:** Drosophila, sleep, feeding, obesity, diabetes, metabolism

## Abstract

One of the brain's most important functions is the control of homeostatically regulated behaviors. Dysregulation of the neural systems controlling sleep and feeding underlies many chronic illnesses. In a recent study published inCurrent Biology we showed that flies, like mammals, suppress sleep when starved and identified the genes Clock and cycle as regulators of sleep during starvation.  Here we show that starvation specifically disrupts sleep initiation without affecting sleep consolidation. The identification of genes regulating sleep-feeding interactions will provide insight into how the brain integrates and controls the expression of complex behaviors.

Sleep and feeding are mutually exclusive behaviors. Consequently, an animal must decide which behavior to express based on internal drives and environmental cues. These behaviors are also functionally interconnected: food-deprivation suppresses sleep, while sleep loss induces hunger [1,2]. Extreme dysregulation of either behavior on its own is deleterious. Longitudinal studies in humans have revealed increased Body Mass Index in short sleeping individuals [3]. The neuropeptides Orexin and neuropeptide Y (NPY) both suppress sleep and promote feeding [4,5], while mice mutant for the leptin receptor have disrupted sleep patterns [6].

Sleep loss potently affects insulin function and has been clinically linked to *Diabetes mellitus, *metabolic syndromes, like *Diabetes mellitus* and obesity. It is possible that the interplay between sleep and metabolic syndromes occurs through the direct effect of sleep on metabolism or indirectly through the dysregulation of appetite [7]. Understanding the molecular and neural link between sleep and feeding will aid in our understanding of obesity and sleep-linked disorders.

Much of the genetic architecture controlling sleep, feeding and metabolism is conserved across phyla. A powerful genetic toolkit has been developed in the fruit fly, *Drosophila melanogaster,* that allows for the manipulation of genes and neural circuits with regional and temporal specificity [8]. Genetic screens in *Drosophila* have led to the identification of many genes affecting sleep, feeding and metabolism with conserved function in mammals. For example the Dopamine transporter promotes sleep [9,10] and a genome-wide obesity screen identified the hedgehog pathway as a conserved determinant of fat generation [11].

To gain insight into the genetic and neural basis of sleep-feeding interactions we investigated the effects of food-deprivation on *Drosophila* sleep. Energy stores and sleep needs are linked suggesting a link between metabolism and sleep [12]. In addition, because starved flies only survive 1-2 days, we reasoned they might be particularly sensitive to the sleep-suppressing effects of food-deprivation.

We therefore monitored flies' activity over a 24-hour period in small tubes with either standard fly food or agar as a feeding substrate (Figure 1A, B). We found that wild-type flies robustly suppress sleep following 12-hours of starvation on agar (Figure 1C, D), suggesting that the effect of food-deprivation on sleep that was previously documented in mammals is conserved in *Drosophila.*

Mammalian sleep is composed of distinct stages that can be characterized by unique electrophysiological properties. Sleep in flies is a also accompanied by alterations in neural activity [13], yet the relevance of these changes to mammalian sleep states remains unclear. Consolidation of sleep can be measured behaviorally in flies by determining the average length and total number of individual sleep bouts. Disruption in bout number suggests difficulty in initiating sleep while shortened bout length indicates a failure to maintain sleep. We found that 24 hours of starvation decreases bout number without affecting bout length (Figure 1E, F). Therefore, food-deprivation specifically affects the onset of sleep without affecting sleep maintenance.

**Figure 1.**
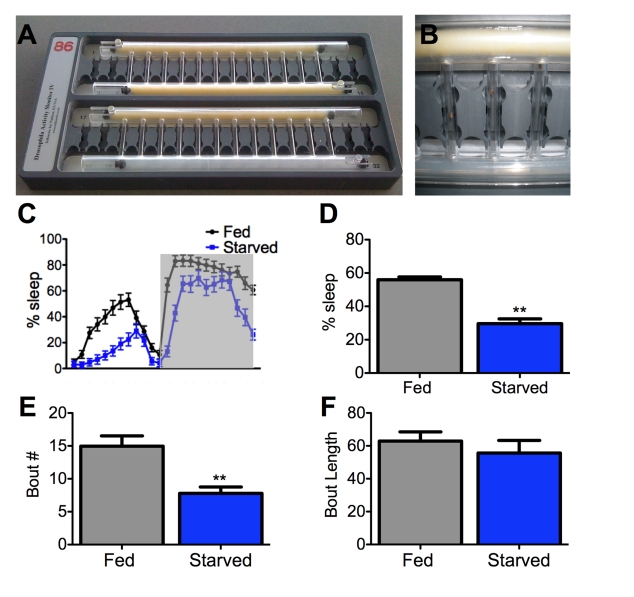
Starvation impairs sleep initiation but not maintenance. (**A**,**B**) A *Drosophila* activity monitor typically used for sleep studies can record up to 32 flies simultaneously. An individual fly is housed in each vertical tube and an infrared beam detects activity. The large horizontal tubes contain either food (yellow) or agar (translucent). Sliding barriers control access to each substrate [32]. Both tubes contain food for fed controls (**A**, top), while agar is provided to the starved experimental group (**A**, bottom; and **B**) on day 2 of testing the experiment (starved, experimental). (**C,D**) Female flies starved for 24 hours sleep less than fed counterparts. Shaded area (**C**) represents lights-off.  (**E**,** F**) The total number of sleep bouts (Bout #) is decreased in starved flies while average bout length does not differ from fed counterparts. Asterisk denotes significant difference (P<0.01, ANOVA) from control groups. Data are mean ± SEM.

We screened for mutants with aberrant sleep during starvation in order to identify genes linking sleep and feeding.  We found that mutants for the genes *Clock* and *cycle *are hypersensitive to the wake-promoting effects of food-deprivation. *Clock* and *cycle *are transcriptional activators that are expressed in ~150 central brain neurons, multiple populations of sensory neurons and peripheral cells. *Clock *and *cycle* function as binding partners and are required for 24-hour transcriptional cycling of the core-circadian clock [14].

In addition to regulating circadian rhythms, *Clock* and *cycle* have been implicated in the regulation of sleep, feeding, olfaction, and starvation resistance [15-18]. *Clock-*dependent modulation of each behavior appears to be conferred through distinct neuronal populations.  For example, *Clock*-regulated control of circadian behavior localizes to eight neurons termed the small ventrolateral neurons [15] while regulation of feeding and starvation resistance localize to the gustatory neurons and fat body bodies [16,18]. Through tissue-specific disruption of *Clock* function we probed populations of cells for their role in starvation-induced sleep suppression. Selectively disrupting *Clock* function in a population of dorsally located neurons in the central brain phenocopied the genetic mutant. However, eliminating *Clock* function in cells previously implicated in circadian locomotor behavior, feeding, olfaction, vision, or starvation-resistance did not affect sleep-suppression during starvation. Therefore, cellular control of sleep-feeding interactions appears to be distinct from those controlling other *Clock*-dependent behaviors.

The pleiotropic nature of behavior suggests many additional genes function in concert with *Clock* and *cycle *to modulate sleep-feeding interactions. Neuropeptide F, the *Drosophila *ortholog of Neuropeptide Y, has been implicated in control of feeding [19] and motivational behavior [20] and is an excellent candidate for modulating sleep-feeding interactions. The mammalian gastrointestinal satiety-inducing peptide cholecys tokinin (CCK) has been reported to induce sleep and a CCK-A receptor antagonist blocks this effect [21]. The function of *drosulfakinin, *the fly ortholog of CCK, is unknown. It is expressed in the brain [22] and represents a candidate for signaling nutrients cues to *Clock*-expressing neurons.

In mammals, hypothalamic Orexin regulates both sleep and feeding and Orexin signaling has been proposed as an attractive drug target for dysfunction of both sleep and feeding systems [23,24]. In addition to Orexin, T-type Ca^2+^ channels have been linked to regulation of sleep-feeding interactions. Administration of a selective T-Type Ca^2+^ channel antagonist increases sleep and reduces body fat in mice fed a high-fat diet [25]. In flies, Ca^2+^ homeostasis has been linked to sleep-wake regulation [26] and future investigation of the role of specific Ca^2+^ channels in the regulation of sleep and feeding may be informative.

Our study focused on the effect of food-deprivation on sleep. The consequences of sleep-deprivation on metabolism were not addressed.  Loss of sleep has detrimental effects on metabolism and has been linked to conditions such as obesity and diabetes [27], and the *Drosophila* insulin-producing cells have been shown to regulate sleep. Hyperexcitation of insulin insulin- producing cells inhibits sleep [28] while activation of the Epidermal Growth Factor Receptor activation in these cells induces sleep [29]. These findings suggest a functional link between the systems controlling insulin and sleep.  Furthermore, alterations in mice mutant for *Clock *and *BMAL1, *the mammalian orthologs of *Clock *and *cycle*, have significant metabolic defects that include decreased insulin release and a diminished ability to maintain normal blood glucose levels [30,31]. Future work examining the metabolism of short-sleeping *Drosophila* mutants may aid our understanding of the link between sleep loss and metabolic dysfunction.

Identifying the molecular basis of behavioral integration will pave the way for the development of drugs that act in a context-dependent fashion. Our findings that *Clock* and *cycle* regulate sleep during food-deprivation is a starting point for understanding the complex interactions regulating sleep and feeding. Utilizing currently available fly mutants to verify candidate genes identified in large-scale fly and mammalian analyses should significantly improve our understanding of sleep-feeding interactions and resulting pathologies.
